# Machine-Learning Methods for Speech and Handwriting Detection Using Neural Signals: A Review

**DOI:** 10.3390/s23125575

**Published:** 2023-06-14

**Authors:** Ovishake Sen, Anna M. Sheehan, Pranay R. Raman, Kabir S. Khara, Adam Khalifa, Baibhab Chatterjee

**Affiliations:** Department of ECE, University of Florida, Gainesville, FL 32611, USA

**Keywords:** neural signals, machine learning, speech recognition, handwriting recognition, signal processing

## Abstract

Brain–Computer Interfaces (BCIs) have become increasingly popular in recent years due to their potential applications in diverse fields, ranging from the medical sector (people with motor and/or communication disabilities), cognitive training, gaming, and Augmented Reality/Virtual Reality (AR/VR), among other areas. BCI which can decode and recognize neural signals involved in speech and handwriting has the potential to greatly assist individuals with severe motor impairments in their communication and interaction needs. Innovative and cutting-edge advancements in this field have the potential to develop a highly accessible and interactive communication platform for these people. The purpose of this review paper is to analyze the existing research on handwriting and speech recognition from neural signals. So that the new researchers who are interested in this field can gain thorough knowledge in this research area. The current research on neural signal-based recognition of handwriting and speech has been categorized into two main types: invasive and non-invasive studies. We have examined the latest papers on converting speech-activity-based neural signals and handwriting-activity-based neural signals into text data. The methods of extracting data from the brain have also been discussed in this review. Additionally, this review includes a brief summary of the datasets, preprocessing techniques, and methods used in these studies, which were published between 2014 and 2022. This review aims to provide a comprehensive summary of the methodologies used in the current literature on neural signal-based recognition of handwriting and speech. In essence, this article is intended to serve as a valuable resource for future researchers who wish to investigate neural signal-based machine-learning methods in their work.

## 1. Introduction

Acquiring and analyzing neural signals can greatly benefit individuals who have limitations in their movement and communication. Neurological disorders, such as Parkinson’s disease, multiple sclerosis, infectious diseases, stroke, injuries of the central nervous system, developmental disorders, locked-in syndrome [1], and cancer, often lead to physical activity impairments [2]. The acquisition of neural signals, along with stimulation and/or neuromodulation using BCIs [3], aims to alleviate some of these conditions. In addition, neural signals have been utilized in various fields such as security and privacy, cognitive training, imaginary or silent speech recognition [4,5], emotion recognition [6,7], mental state recognition [8], human identification [9,10], speech communication [11], synthesized speech communication [12] gaming [13], Internet of Things (IoT) applications [14], Brain Machine Interface (BMI) applications [15,16,17], neuroscience research [18,19], speech activity detection [20,21] and more. The first step involves collecting neural signals from patients, which are then processed and analyzed. The processed signals are then used to operate assistive devices, which helps patients with their movements and communication. Neural signals can also be utilized to gauge the mental state of the general population, detect brain injuries or sleep disorders, and identify individual emotions [22]. Speech-based BCIs have shown great potential in assisting patients who have experienced brainstem strokes or amyotrophic lateral sclerosis (ALS) and are consequently diagnosed with Locked-in Syndrome (LIS). These patients can only interact with others using restricted movements, such as eye movements or blinking [23]. Again, BCI technology can assist in facilitating high-performance communication with individuals who are paralyzed [24,25]. Additionally, speech BCI can be beneficial for individuals suffering from aphasia, a condition that causes pathological changes in cortical regions related to speech [26]. Researchers are currently developing intracortical BCI to aid individuals with motor disabilities in their communication and interaction with the environment [27]. However, this technology relies on recordings from the primary motor cortex, which can potentially exhibit day-to-day variability [28].

Neural signals can be collected using two primary methods: invasive and non-invasive. Using the invasive method, signals (for example, Electrocorticogram (ECoG) [29,30]) are collected from inside the skull, which requires surgery. On the other hand, in the non-invasive method, signals, such as Electroencephalogram (EEG) [31,32], are collected from the scalp, which does not require surgery. However, the amplitude of the signals that are received using non-invasive methods is usually smaller than signals received with invasive techniques. Nevertheless, signal acquisition using non-invasive methods is easier and safer than invasive techniques, and because of that there is a strong research interest in improving the signal-to-noise ratio (SNR) [33] in non-invasive methods using specific signal processing techniques. Both invasive and non-invasive signals can be used to detect brain patterns and help individuals recognize handwriting, speech [34], silent speech [35], emotion, and mental states. Another widely recognized neural signal extensively employed in the field of BCI is the steady-state visual-evoked potential (SSVEP). SSVEP refers to a measurable, objective fluctuation of electrical brain activity triggered by a particular group of visual stimuli. SSVEP provides a stable and consistent neural response to visual stimuli. SSVEP can be detected using non-invasive techniques, e.g., EEG technology. SSVEP-based systems provide high information transfer rates. Again, different visual targets or objects provide different SSVEP responses. SSVEPs are used for implementing EEG-based BCI spellers as well [36].

The BCI technology related to detecting handwriting and speech from neural signals is a new research area. Individuals with severe motor impairments can greatly benefit from this type of BCI technology as it can significantly enhance their communication and interaction capabilities [37]. Consequently, there is a growing demand for research in this field. This paper aims to provide readers with an overview of the existing research conducted on the recognition of handwriting and speech from neural signals up to this point.

This review summarizes articles that have used both invasive and non-invasive signals to detect handwriting as well as speech. To the best of our knowledge, this is the first review paper that involves both of these applications with acquired neural signals. At the same time, we try to draw important conclusions such as (1) what are the regions of the brain that is responsible for generating the intended neural signals, and where the electrodes should be placed to achieve larger signal-to-noise ratio; (2) apart from speech and handwriting detection, what are the other applications that can be enabled with similar signal acquisition and processing techniques; and (3) which machine learning models are becoming more popular for such scenarios and why. Individuals who have lost their speaking or movement/writing capabilities require external support, and advancements in this field can be extremely beneficial to the society as well as researchers interested in neural signal processing.

## 2. Regions of the Brain Responsible for Handwriting and Speech Production

The production of speech involves several stages in the brain, including the translation of thoughts into words, the construction of sentences, and the physical articulation of sounds [38]. Three key areas of the brain are directly involved in speech production: the primary motor cortex, Broca’s area, and Wernicke’s area [39]. Wernicke’s area is primarily responsible for producing coherent speech that conveys meaningful information. Damage of Wernicke’s area, also known as fluent aphasia, can affect comprehension and meaningless sentences [40]. Broca’s area aids in generating smooth speech and constructing sentences before speaking. Damage to one’s Broca’s area results in a condition known as Broca’s aphasia, or non-fluent aphasia, which can cause the person to lose their ability to produce speech sounds altogether or to only speak slowly and in short sentences [39]. Finally, the motor cortex plays a role in planning and executing the muscle movements necessary for speech production, including the movement of the mouth, lips, tongue, and vocal cords. Damages to the primary motor cortex can cause paralysis of the muscles used for speaking. However, therapy and repetition can help improve these impairments [41].

**Figure 1:**
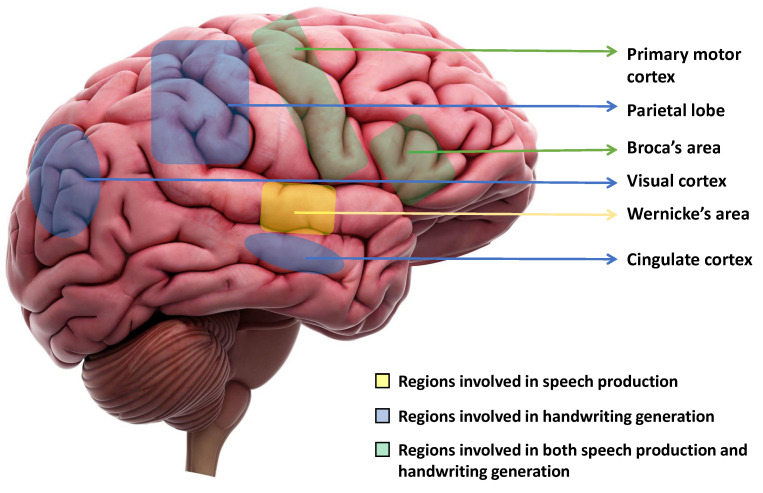
Key regions of the brain that are fundamentally responsible for speech production and initiating motor movements for generating handwriting. Wernicke’s area is responsible for speech production. The parietal lobe, Visual cortex, and Cingulate cortex are responsible for handwriting generation. The primary motor cortex and Broca’s area are responsible for both speech production and handwriting generation.

When writing is initiated, our ideas are first organized in our mind, and the physical act of writing is facilitated by our brain, which controls the movements of our hands, arms, and fingers [42]. This process is initiated by the cingulate cortex of the brain. The visual cortex then creates an internal picture of what the writing will look like. Next, the left angular gyrus [43] converts the visual cortex signal into a comprehension of words, and this process involves Wernicke’s area also. Finally, the parietal lobe and the primary motor cortex work together to coordinate all of these signals and produce motor signals that control the movements of the hand, arm, and finger required for writing [42]. In a study, Willett et al. [44] proposed a discrete BCI, which is capable of accurately decoding limb movements, including those of all four limbs, from the hand knob [45,46]. Figure 1 shows the regions of the brain that are primarily responsible for speech production and motor movements for handwriting.

## 3. Methods of Collecting Data from Brain

**Figure 2:**
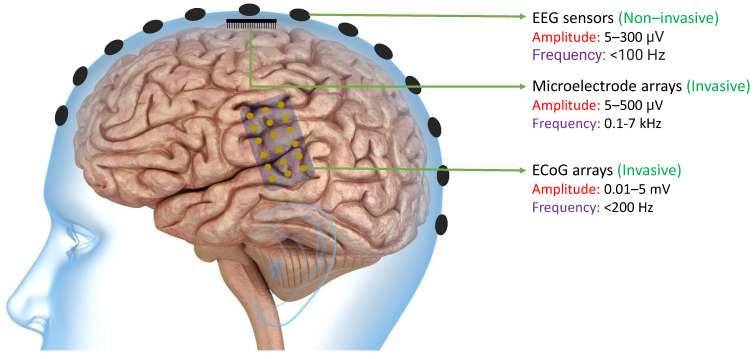
Existing technologies like EEG Sensors, ECoG Arrays, and Microelectrode Arrays that are used to acquire neural signals with their acquired signal characteristics including amplitude and frequency bands [47]. The amplitudes of neural signals acquired from ECoG arrays and the frequency of neural signals acquired from microelectrode arrays are typically higher than other existing technologies.

The primary objective of many BCIs is to capture neural signals in a manner that allows external computer software or devices to interpret them with ease. Neural signals can be obtained from the brain through various methods such as EEG sensors, Microelectrode arrays, or ECoG arrays. As shown in Figure 2, EEG signals can be extracted non-invasively from the scalp. because of which they typically have lower magnitudes compared to other neural signals. On the other hand, ECoG arrays can produce signals of higher magnitude since they are implanted invasively in the brain. However, because of their physical dimensions, the spatial resolution is still limited. Finally, microelectrode arrays can acquire high-frequency spikes with much improved spatial resolution [47]. In all of these methods, the signals must be processed in a way that enables the BCI software or devices to effectively decipher them [48].

### 3.1. Invasive Methods

The neural signals are directly collected using invasive electrodes, placed inside the skull. Here, brain surgery is needed for implanting the electrodes into the grey matter of the brain. As the signals are coming directly from the grey matter of the brain this technique always provides high-quality signals [49], with better SNR. However, as it requires surgery for implanting the electrodes inside the skull, the invasive methods carry a high risk of brain infection. Additionally, in invasive methods, the brain reacts with a process called gliosis that creates scar tissue around the foreign object (electrode), and thus the electrodes can hardly collect neural signals [50] over time. Most of the papers included in this review that utilizes invasive methods, have extracted the signals from the primary motor cortex area of the brain [51].

**Figure 3:**
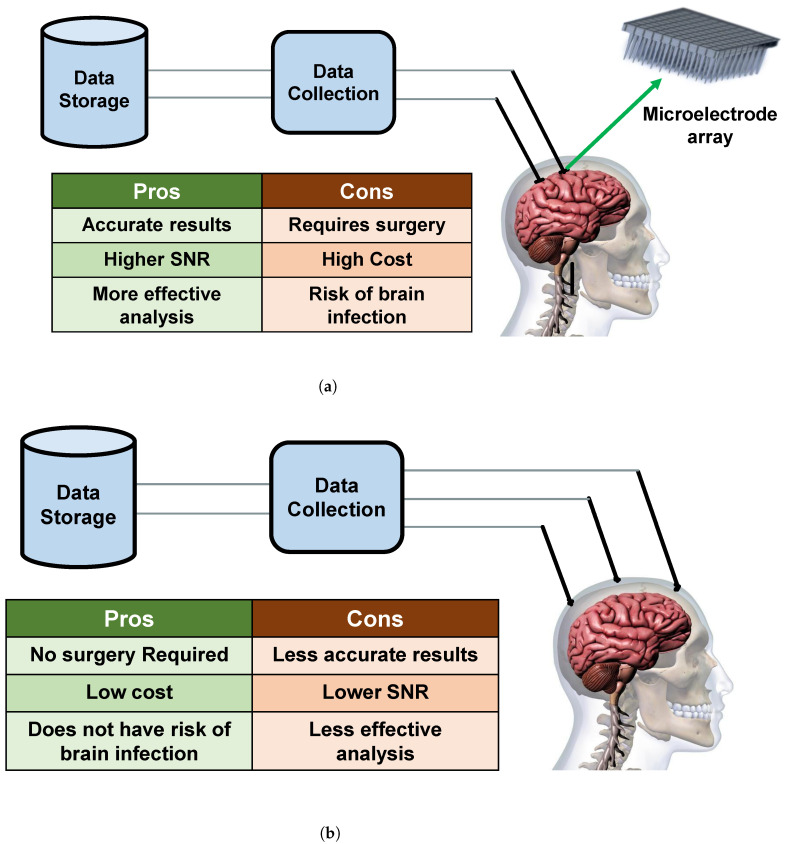
Existing methods of collecting neural signals from brain. (**a**) Data processing flow diagram, advantages, and disadvantages of invasive process of collecting neural signals from the brain. Though invasive process requires surgery and high cost, neural signals that are extracted from invasive process provide accurate results and higher SNR. (**b**) Data processing flow diagram, advantages, and disadvantages of non-invasive process of collecting neural signals from the brain. The non-invasive process requires no surgery and low cost, but the neural signals acquired from the non-invasive process provide less accurate results and lower SNR.

Invasive methods of collecting neural signals are mostly used in medical applications in a hospital setting. As the signals are more accurate, they can be used to help paralyzed patients with certain functionalities to move or provide commands through computers. Since there is direct contact with neurons at the time of collecting signals they provide more information even if the signals are coming from only a few neurons. These signals can be used to control artificial arms [52], speech decoding [53], TV, lights, Brain to Text implementation [54], speech recognition [55,56] and other software applications [57]. Figure 3a shows the invasive process of collecting the invasive signals from the brain.

### 3.2. Non-Invasive Methods

In the non-invasive way of collecting neural signals, the electrodes are placed on the scalp/skin to measure and collect neural signals. This technique has been used widely because it’s easier to use, and does not require surgery as the neural signals are acquired using external sensors or electrodes. Hence, it is cheaper and provides more comfort to the person and it is also less risky.

However, as the signals are collected at a larger distance from the actual neurons, it provides noisy data and worse signal resolution. Thus, this method is less effective than the invasive methods in terms of the SNR. Most of the non-invasive ways focus on collecting EEG signals as it is easier and cheaper. However, the EEG signals can vary from person to person, and even within the subject from time to time [58]. Therefore, it is very difficult to deal with the real-time experiment that the model has trained with the past EEG signals dataset [59].

In the non-invasive techniques, the neural signals can also be sent back into the brain using transcranial magnetic stimulation (TMS) which has already been used by medics [49]. EEG signals are also used to recognize unspoken [60] and imagined speech from individuals [61,62]. Examples of non-invasive techniques are EEG [63], magneto-encephalography (MEG) [64], functional magnetic resonance imaging (fMRI) [65], and near-infrared spectroscopy (NIRS) [66]. Figure 3b shows the non-invasive process of collecting EEG data from the brain.

## 4. Articles Related to Handwriting and Speech Recognition Using Neural Signals

### 4.1. Speech Recognition Using Non-Invasive Neural Datasets

In 2017, Kumar et al. [67] proposed a Random Forest (RF) based silent speech recognition system utilizing EEG signals. They introduced a coarse-to-fine-level envisioned speech recognition model using EEG signals, where the coarse level predicts the category of the envisioned speech, and the finer-level classification predicts the actual class of the expected category. The model performed three types of classification: digits, characters, and images. The EEG dataset comprised 30 text and non-text class objects that were imagined by multiple users. After performing the coarse-level classification, a fine-level classification accuracy of 57.11% was achieved using the Random Forest classifier. The study also examined the impact of aging and the time elapsed since the EEG signal was recorded.

In 2017, Rosinová et al. [68] proposed a voice command recognition system using EEG signals. EEG data were collected from 20 participants aged 18 to 28 years, consisting of 13 females and 7 males. The EEG data of 50 voice commands were recorded 5 times during the training phase. The proposed model was tested on a 23-year-old participant, whose EEG signal data was collected when speaking the 50 voice commands 30 times. The hidden Markov model (HMM) and Gaussian Mixture model (GMM) were used to train and test the proposed model. The authors claim that the highest classification accuracy was achieved on alpha, beta, and theta frequencies. However, the recording data were insufficient and the accuracy was very low.

In 2019, Krishna et al. [69] presented a method for automatic speech recognition from an EEG signal based on Gated Recurrent Units (GRU). Their proposed method was trained on only four English words—“yes”, “no”, “left”, and “right”—spoken by four different individuals. The proposed method can effectively detect speech in the presence of background noise, with a 60 dB noise level used in the research. The paper reported a high recognition accuracy of 99.38% even in the presence of background noise.

In 2020, Kapur et al. [35] proposed a silent speech recognition system based on Convolutional Neural Network (CNN) utilizing neuromuscular signals. This research marks the first non-invasive real-time silent speech recognition system. The dataset used comprised 10 trials of 15 sentences from three multiple sclerosis (MS) patients. The research obtained 81% accuracy, and an information transfer rate of 203.73 bits per minute was recorded.

In 2021, Vorontsova et al. [2] proposed a silent speech recognition system based on Residual Networks (ResNet)18 and GRU models that use EEG signals. The researchers collected EEG data from 268 healthy participants who varied in age, gender, education, and occupation. The study focused on the classification of nine Russian words as silent speech. The dataset consists of a 40-channel EEG signal recorded at a 500 Hz frequency. The results showed an 85% accuracy rate for the classification of the nine words. Interestingly, the authors found that a smaller dataset collected from a single participant can provide higher accuracy compared to a larger dataset collected from a group of people. However, the out-of-sample accuracy is relatively low in this study.

### 4.2. Speech Recognition Using Invasive Neural Datasets

In 2014, Mugler et al. [70] published the first research article about decoding the entire set of phonemes from American English. In linguistics, a phoneme refers to the smallest distinctive unit of sound in a language, which can be used to differentiate one word from another [71]. The authors used ECoG signals from four individuals. In this study, a high-density (1–2 mm) electrode array with 4 cm of speech motor cortex was used to decode speech. The researchers achieved 36% accuracy in classifying phonemes using ECoG signals with Linear Discriminant Analysis (LDA). However, the accuracy in word identification from phonemic analysis alone was only 18.8%, which falls short of the mark.

In 2019, Anumanchipalli et al. [72] proposed a speech restoration technique that converts brain impulses into understandable synthesized speech at the rate of a fluent speaker. Bidirectional long short-term memory (BLSTM) was used to decode kinematic representations of articulation from high-density ECoG signals collected from 5 individuals.

In 2019, Moses et al. [73] proposed a real-time question-and-answer decoding method using ECoG recordings. The authors used the Viterbi decoding algorithm which is the most commonly used decoding algorithm for HMM. The real-time high gamma activity of the ECoG signals has been collected from the brain. The authors received 61% decoding accuracy for producing utterances and 76% decoding accuracy for perceiving utterances.

In 2020, Makin et al. [74] published an article on machine translation of cortical activity to text using ECoG signals. The authors trained a Recurrent Neural Network (RNN) to encode each sentence-length sequence of neural activity. The encoder-decoder framework was employed for machine translation. The authors decoded cortical activity to text based on words, as they are more distinguishable than phonemes. For training purposes, 30–50 sentences of data were used.

In 2022, Metzger et al. [75] proposed an Artificial Neural Network (ANN) based model for recognizing attempts at silent speech mainly built on GRU layers. ECoG activity from the neural signal, along with a speech detection model, was used for spelling sentences. Only code words from the North Atlantic Treaty Organization (NATO) phonetic alphabet [76] were used during spelling to improve the neural discriminability from one word to another. In online mode, an 1152-word vocabulary model was used, with a 6.13% character error rate and 29.4 characters per minute. The beam search technique was used to spell the most accurate sentences. However, only one participant was involved in this training and spelling process.

### 4.3. Handwritten Character Recognition Using Non-Invasive Neural Datasets

In September 2015, Chen et al. [77] proposed a BCI speller using EEG. The study implemented a Joint Frequency Phrase Modulation (JFPM) based SSVEP speller to achieve high-speed spelling. Eighteen participants took part in the study, and six blocks of 40 characters were used for training with 40 trials on each block in random order. The study found a spelling rate of up to 60 characters per minute and an information transfer rate of up to 5.32 bits per second.

Saini et al. [78] presented a method for identifying and verifying individuals using their signature and EEG signals in 2017. The study involved collecting signatures and EEG signals from 70 individuals between the ages of 15 and 55. Each participant provided 10 signature samples, and EEG signals were captured using an Emotiv Epoc+ neuro headset. The researchers used 1400 samples of signature and EEG signals for user identification, and an equal number of samples for user verification. They evaluated the performance of the method using three types of tests: using only signatures, using only EEG signals, and using signature-EEG fusion. The results showed that the signature-EEG fusion data achieved the highest accuracy of 98.24% for person identification. For user verification, the EEG-based model performed better than the signature-based model and the signature-EEG fusion data. The authors also found that individuals between the ages of 15 and 25 had higher identification accuracy than others, and males had higher identification accuracy than females.

In 2019, Kumar et al. [79] proposed a novel user authentication system that utilizes both dynamic signatures and EEG signals. The study involved collecting signatures and EEG signals from 58 individuals who signed on their mobile phones simultaneously. A total of 1980 samples of dynamic signatures and EEG signals were collected, with EEG signals being recorded using an Emotiv EPOC+ device and signatures being written on the mobile screen. To train the system, a BLSTM neural network-based classifier was utilized for both dynamic signatures and EEG signals. The results showed that the signature-EEG fusion data using the Borda count fusion technique achieved an accuracy of 98.78%. The Borda count decision fusion verification model was used for user verification, which resulted in a false acceptance rate of 3.75%.

In 2021, Pei et al. [80] proposed a method for mapping scalp-recorded brain activities to handwritten character recognition using EEG signals. In the study, five participants provided their neural signal data while writing the phrase “HELLO, WORLD!” CNN based classifiers were employed for the analysis. The accuracy of handwritten character recognition varied among participants, ranging from 76.8% to 97%. The accuracy of cross-participant recognition ranged from 11.1% to 60%.

### 4.4. Handwritten Character Recognition Using Invasive Neural Datasets

In 2021, Willett et al. [81] proposed a brain-to-text communication method using neural signals from the motor cortex. The authors employed a RNN for decoding the text from the neural activity. The proposed model decoded 90 characters per minute with 94.1% raw accuracy in real-time and greater than 99% accuracy offline using a language model. Sentence labeling was performed using a HMM, and the Viterbi search technique was employed for offline language modeling. The authors also demonstrated that handwriting letters with neural activity is easier to distinguish than point-to-point movements.

**Figure 4:**
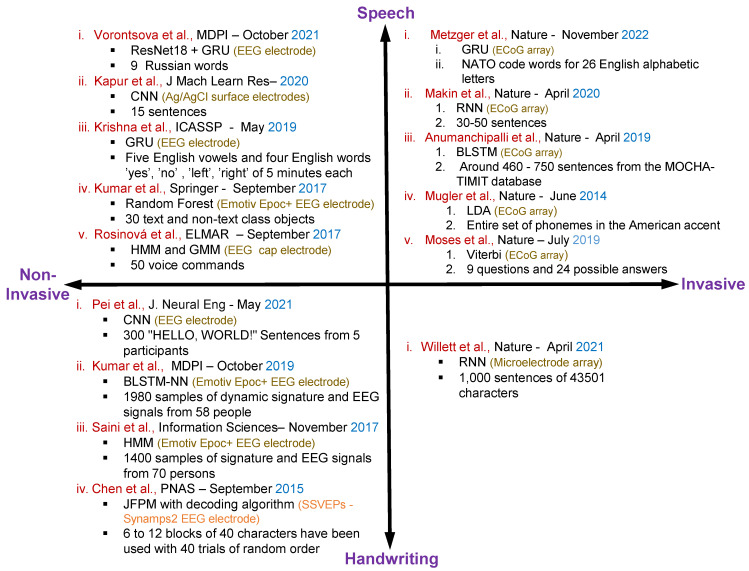
Summary of the existing articles on speech and handwritten character recognition with invasive and non-invasive neural signal acquisition including methods, datasets, electrodes specification and publication details of the individual articles [2,35,67,68,69,70,72,73,74,75,77,78,79,80,81].

Figure 4 shows the overall summary for the speech and handwritten character recognition-based articles with invasive and non-invasive neural signal acquisition.

## 5. General Principle of Using Machine Learning Methods for Neural Signals

The research conducted on neural signals typically follows a standardized flowchart. It begins with the acquisition of neural signals and concludes with the identification of these signals using the most efficient methods. In this context, we will focus on research conducted using machine learning and classical techniques.

**Figure 5:**
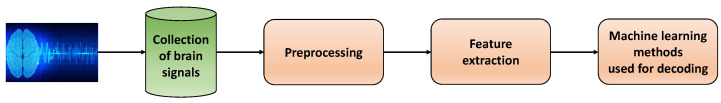
Diagram of data processing and machine learning methods used for decoding neural signals (each block corresponds to one step of the whole process).

Figure 5 depicts a step-by-step diagram commonly utilized in existing research articles that work with neural signals. To begin, invasive or non-invasive processes are used to collect, digitize and store neural signals. These signals then undergo a series of preprocessing techniques to enhance their quality. Next, meaningful features are extracted from the processed signals. Finally, machine learning methods are employed to accurately decode the signals. The various steps involved in the research articles have been summarized in the following subsections. 

### 5.1. Prepossessing Techniques and Feature Extraction Methods

Most of the papers used independent component analysis and principle component analysis in their preprocessing stages. For extracting meaningful features from the raw data the authors used Mel Frequency Cepstrum Coefficients (MFCCs) in most of the papers for recognizing speech or silent speech. In [81], the authors labeled the sentences using a hidden Markov model. They provided a neural representation of the attempted handwriting using principal component analysis and time-warping of the neural activity. Additionally, they showed a 2D visualization of the neural activity using t-distributed stochastic neighbor embedding. In [80], the EEG signals were first downsampled to 250 Hz and bandpass filtered between 1 and 45 Hz. Additionally, silent parts of the signals were removed. Next, Independent Component Analysis (ICA) was applied to extract meaningful features. In [78], the raw EEG signals are smoothed using the Moving Average (MA) filter and then Discrete Wavelet Transform (DWT) analysis has been applied for decomposing the signals. Furthermore, features from the gamma frequency band were measured from the EEG signals. In a separate article [79], DFT features were extracted from EEG signals for use in user authentication. In the case of dynamic signatures, the feature generation process involved combining the signature trajectory and writing direction, which were both measured. In [73], Several preprocessing techniques like amplification, quantization, noise removal, and sampling have been performed on the raw ECoG data. The PCA-LDA model have been also used here for extracting principle components.

In [75], the neural signals were first digitized using a percutaneous pedestal connector. Next, noise cancellation and anti-aliasing filters were applied to the signals, which were streamed at 1 kHz. In [72], Dynamic Time Warping (DTW) was used in conjunction with Mel Frequency Cepstral Coefficients to extract important features from silent speech. In [70], the ECoG signals were marked according to the onset of phoneme time, and Fast Fourier Transform (FFT) was performed on the ECoG signal. This was done to convert the signals into meaningful features by combining FFT coefficients to form each frequency band of interest. In [69], the first and most important 13 MFCC features were extracted, and first and second-order differentials were computed. This resulted in a total of 39 MFCC features, which were sampled at 100 Hz and mainly used for training purposes. The raw EEG signals are first processed using a moving average filter to remove various types of noise, trends, and artifacts in [67]. Next, the Standard Deviation, Root Mean Square, Sum of Values, and Energy of the signals are computed to extract features. In [35], heartbeat artifacts and high-frequency noise were removed from the Surface Electromyography (sEMG) signals, which were then sampled at 1 kHz. In [68], the raw EEG signals were first normalized, and then a 2nd-order Butterworth band-stop, low-pass, and high-pass filter was used to remove muscular artifacts and random noise.

### 5.2. Features of the Brain Signals Used in Existing Research

For detecting handwriting and speech from neural signals different types of features have been used in the existing research. Neural features are highly classified by the way of extracting neural signals from the brain, i.e., invasively, or non-invasively. The most used frequency bands and their approximate spectral boundaries of EEG signals are delta (1–3 Hz), theta (4–7 Hz), alpha (8–12 Hz), beta (13–30 Hz), and gamma (30–100 Hz) [82]. For ECoG signals the commonly used frequency bands and their most approximate spectral boundaries are gamma (30–70 Hz) and high-gamma (>80 Hz) [83]. Table 1 shows the summary table for features used in the existing research.

### 5.3. Machine Learning Methods Used for Training Neural Signals

**Figure 6:**
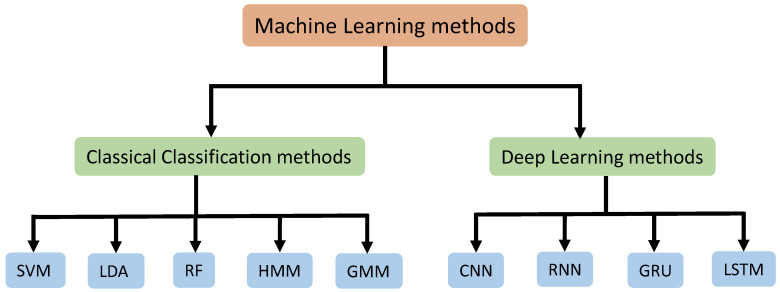
In this review, the machine learning methods that have been used in the existing research are divided into Classical Classification methods and Deep Learning methods to illustrate the existing research more clearly. SVM, LDA, RF, HMM, and GMM fall under classical classification methods, and CNN, RNN, GRU, and LSTM fall under deep learning methods.

The machine learning methods used for training neural signals have been divided into 2 parts namely classical classification methods and deep learning methods. We summarized the methods used in the existing research that worked with neural signals. The Figure 6 shows our methods division strategy for better understanding.

#### 5.3.1. Classical Classification Methods

Several studies have utilized classical models to train neural signals for recognizing both speech and handwriting activities. The majority of these studies have employed HMM and GMM to train brain activities. One article [68] used HMM and GMM to train and test EEG signals obtained from the brain. In another study, authors in [78] employed sequential HMM for evaluating three types of testing, including testing with only signatures, testing with only EEG signals, and testing with signature EEG fusion. In [73], the authors used the Viterbi decoding algorithm which is one of the most useful and commonly used decoding algorithms for HMM.

In addition to HMM, LDA was used in [70] to train the entire set of American English phonemes from the ECoG signal. Lastly, JFPM along with a decoding algorithm has been used which utilized SSVEPs [36,77] to implement an EEG-based BCI speller.

In [67], the authors have proposed a classifier based on RF that operates at both a coarse and fine level. To identify three distinct levels of classes, three RF classifiers were run in parallel. The authors stated that the RF classifier is superior to SVM and ANN-based classifiers because it employs bagging ensemble and bootstrap aggregation techniques to create multiple models that are combined to yield greater accuracy.

**Figure 7:**
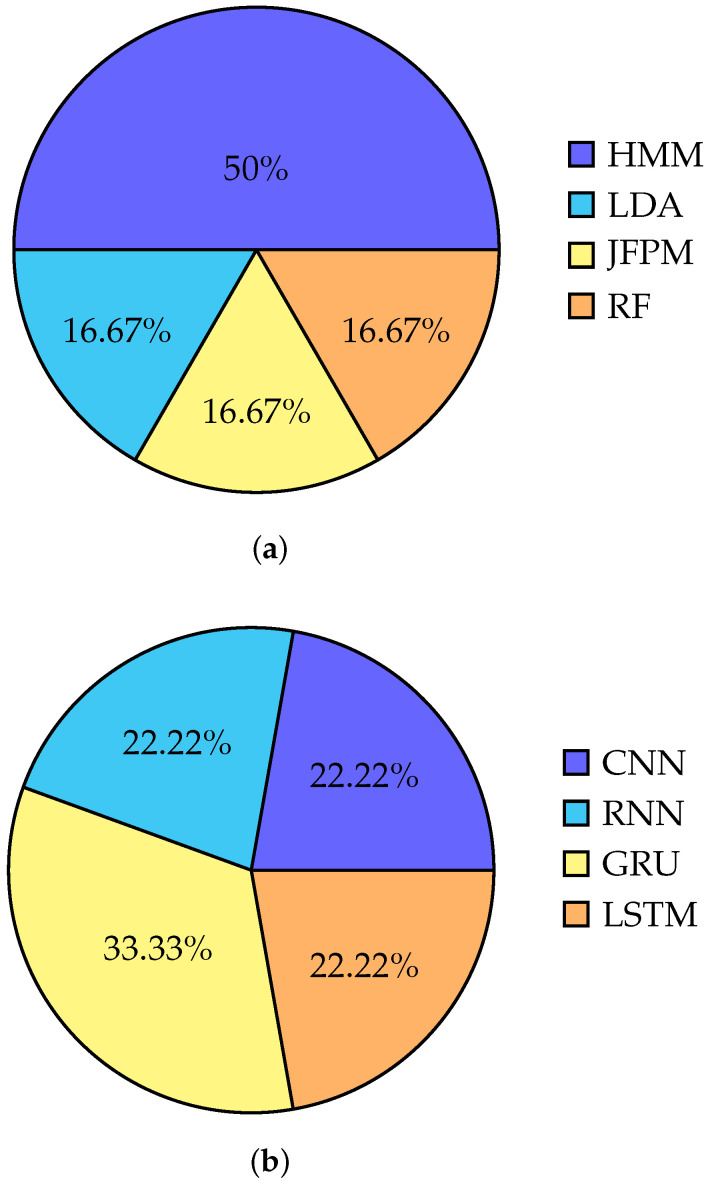
Pie charts of deep learning and classical methods used in existing research for speech and handwriting detection from neural signals. (**a**) Pie chart of deep learning methods used. This pie chart visualizes that GRU is dominating than CNN, RNN, and LSTM in this research field. (**b**) Pie chart of classical classification methods used. This pie chart visualizes that HMM is dominating in this research field as a classical classification method.

Figure 7a shows the overall accuracy of classical classification methods used till now for working with neural signals.

#### 5.3.2. Deep Learning Methods

Most recent articles have employed machine learning techniques to decode EEG signals from the brain [84]. Again, machine learning methods have been used in the training phase of most of the papers. Here the neural data have been trained and tested using various machine learning models. Most of the researchers use RNN for developing the model because of the ability of RNNs to process time-series data better. However, in certain scenarios, CNN is used at the time of training the model with the neural dataset.

In [81], RNNs were used to convert the neural activity into probabilities that describe the likelihood of characters that will be written. The probabilities are then thresholded to identify the actual character. Again for decoding sentences to words from ECoG signals. In [74], an encoder RNN was used to encode each sentence span of neural signal into a conceptual expression. Then, a decoder RNN was used to decode this expression into words and English sentences.

The GRU is also commonly used in most silent speech recognition tasks that involve non-invasive neural signals. In [69], a GRU-based deep learning model was trained using three different feature sets, including only EEG features, only acoustic features, and the concatenation of acoustic and EEG features. In [2], the authors achieved the best results using a ResNet18 + 2GRU neural network. They did not use any dropout, and the Adam optimizer was employed with a 16-mini batch size and a 0.01 learning rate.

BLSTM neural network-based models have also been utilized for a variety of tasks, including speech and handwriting recognition from neural signals. In the article previously discussed [79], a BLSTM neural network-based classifier was employed for both dynamic signatures and EEG signals, both individually and in combination. Here [85], a deep Long Short Term Memory (LSTM) has been used to recognize imaginary speech from EEG data. In another article [72], BLSTM was utilized for decoding kinematic representations of articulation from ECoG signals.

CNN models were also used in the training process. In [35], a CNN model with 5-fold repeated stratified cross-validation was trained using the Adam optimizer and a batch size of 50 to minimize the cross-entropy loss of the spoken dataset. To recognize imaginary speech from EEG data CNN has been used with cross-validation [4]. In [80], the 2D ERP, pattern segments are processed and identified as images, which are then trained on a CNN model to achieve higher accuracy. In [86], a densely connected 3D CNN has been used for speech synthesis from ECOG signals.

The authors in [75] have developed an artificial neural network for speech detection and letter classification. The neural network includes a 1D CNN input layer, followed by two layers of bidirectional GRU. This configuration was chosen to optimize accuracy in these tasks. The authors in [72], employed BLSTM to convert recorded cortical activity into articulatory movement representations, and then converted those representations into speech acoustics during the training process. This approach was utilized to decode cortical activity and improve the accuracy of speech representation. In a study, Hinton et al. [87] proposed a deep neural network-based speech recognition system that outperforms GMMs on a variety of speech recognition benchmarks. Figure 7b shows the distribution of the deep learning methods used in speech and handwritten recognition from neural signals.

## 6. Chronological Analysis of Methods Used for Training Neural Signals

**Figure 8:**
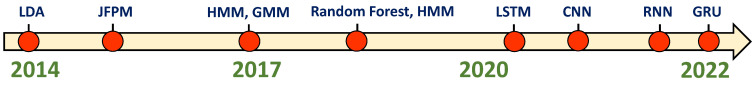
Chronological analysis of techniques used in neural data processing from 2014 to 2022. Classical classification methods were used to dominate in the early stages of this research area but nowadays deep learning methods are dominating in this research field.

Figure 8 presents a chronological overview of the methods used to process the neural signals. Earlier in 2014, researchers employed classical methods such as LDA, SSVEPS, and HMMs to train neural signals. However, over time, machine learning classifier algorithms such as random forests and CNN became more popular for classifying neural signals. In recent years, with the rapid development of ANN, researchers have discovered that advanced RNN architectures, such as LSTM, RNN, and GRU can work better with time-series data such as neural signals. As a result, they have increasingly utilized such networks to train with neural signals from 2020 to the present day. Compared to previous methods used with neural datasets, researchers have achieved higher accuracy working with advanced RNN architectures.

## 7. Discussion

Previous studies have shown that neural signals can assist individuals with disabilities in their communication and movement. Moreover, neural signals have been applied in a variety of fields such as security and privacy, emotion recognition, mental state recognition [88], user verification, gaming, IoT applications, and others. As a result, the research on neural signals is steadily increasing. Although classical methods were once widely used, machine-learning techniques have yielded promising results in recent years.

When working with neural signals, collecting and processing them can be one of the most challenging tasks. As a result, much of the research in this field has been conducted using non-invasive neural signals, which are easier to collect and process. However, some research has also been done on invasive neural signals. Table 2 summarizes the existing research by presenting the dataset, methods, and other important features of each corresponding study. In [89], Nieto et al. also proposed an EEG-based dataset for inner speech recognition. The use of neural signals to recognize a person’s handwriting and speech has received significant attention in recent times. According to a study conducted by authors in [81], identifying letters through neural activity is more practical than point-to-point movements. Inner speech recognition through neural signals is also becoming more popular in research [89].

Most studies on speech-based Brain–Computer Interfaces (BCIs) have used acute or short-term ECoG recordings, but in the future, the potential of long-term ECoG recordings and their applications could be explored further [23]. Currently, the development of high-speed BCI spellers is one of the most popular research directions. Ongoing innovations aim to increase electrode counts by at least an order of magnitude to improve the accuracy of extracting neural signals. Multimodal approaches using simultaneous EEG or ECoG signals to identify individuals have also gained considerable attention in recent years [79]. The performance of BCI communication can be enhanced by applying modern machine learning models to a large, accurate, and user-friendly dataset. In the future, more robust features may be extracted from EEG or ECoG signals to improve system recognition performance.

EEG used to monitor the electrical activity of the brain, is an invaluable tool for investigating disease pathologies. It involves analyzing the numerical distribution of data and establishing connections between brain signals (EEG) and other biomedical signals. These include the electrical activity of the heart measured by electrocardiogram (ECG), heart rate monitoring using a photoplethysmography (PPG), and the electrical activity generated by muscles recorded through electromyography (EMG) [90,91,92]. The integration of neural signals with other biomedical signals has led to diverse applications, such as emotion detection through eye tracking [93], video gaming and game research [94], epilepsy detection [95], and motion classification utilizing sEMG-EEG signals [96,97], among others [98,99].

One other extremely important consideration is the ability to detect and analyze the neural signals in real-time for the production of speech and handwriting. To develop real-time BCI applications, several issues and challenges have to be addressed. The neural data collection methods need to become faster as well as more accurate. The pre-processing techniques for the neural signals should also be improved in terms of their latency and efficiency. At the same time, the decoding and classification methods used on these processed data should also work with good accuracy and low latency. Moreover, for developing real-time BCI, certain features of the neural signals should be extracted from the processed data within a short time. Intraoperative mapping using high-resolution ECoG can be used to produce results within minutes but still, more work need to be done to perform this in real time [100]. The amplitude of the neural signals should remain high, and the latency should remain low. For developing real-time speech detection from ECoG signals the high gamma activity feature has been used in [73]. Again, kinematic features have been used in [81] from ECoG data as well as from EEG [80]. The real-time functional cortical mapping may be used for detecting handwriting and speech from ECoG recording in real time [101]. A Pyramid Histogram of Orientation Gradient features extracted from signature images can be used for fast signature detection from EEG data. Event-related desynchronization/synchronization features from the EEG data may be used for handwriting detection when an individual thinks about writing a character, as shown in [102].

As these technologies target providing access to the signals generated by the brain, ethical issues have emerged regarding the use of BCIs to detect speech and handwriting from neural signals. It is important to consider individuals’ freedom of thought in BCI communication, as modern BCI communication techniques raise concerns about the potential for private thoughts to be read [5]. Key concerns involve the invasion of privacy and the risk of unauthorized access to one’s thoughts. To address these concerns, solutions may include the implementation of regulations, acquiring informed consent, and implementing strong data protection measures. Furthermore, advancements in encryption and anonymization techniques play a crucial role in ensuring the privacy and confidentiality of individuals. Ongoing research endeavors focus on enhancing BCI accuracy and dependability through the development of signal processing algorithms and machine learning models [103,104].

The future of BCI research in detecting handwriting and speech from neural signals shows immense potential. It offers the possibility of improving the lives of individuals with speech or motor impairments by providing alternative communication options. However, there are challenges that need to be overcome, including improving the accuracy and reliability of BCI systems, developing effective algorithms for decoding neural signals, and addressing ethical concerns such as privacy protection. Moving forward, efforts need to be focused by the new researchers in this field on refining signal processing techniques, exploring novel approaches to recording neural activity and advancing machine learning algorithms [105,106]. One other direction that the current research is focused on is the collection of signals using distributed implants [107,108,109,110,111], which can provide simultaneous recording from multiple sites scattered throughout the brain. Such technologies hold immense promise in terms of providing more information from various regions which potentially produces correlated neural activity during the generation of speech and handwriting.

## 8. Conclusions

The future of research in BCIs focusing on the detection of handwriting and speech from neural signals holds significant promise. Innovative advancements in this field have the potential to create a user-friendly and interactive platform that facilitates communication for individuals who experience disabilities related to their mobility, speech, or ability to communicate effectively. In this review paper, we have investigated how the brain signals are generated at the time of speech and the generation of handwriting and the signal collection strategies from the brain. We tried to gather the existing machine learning methods and decoding techniques that work with detecting speech and handwriting from neural signals. We have also investigated which features of the neural signals are very important for recognition purposes. However, to enhance the accuracy of this field, researchers should strive to identify effective signal processing techniques, employ appropriate data collection methods, and select precise machine learning and decoding algorithms suitable for analyzing neural signals.

As non-invasive BCI carries less risk than invasive BCI, research on non-invasive BCI is growing day by day. However, the signals received from non-invasive BCI are weak and prone to interference. Additionally, measuring neural signals is a challenging task. The BCI system is generally much more complicated than other systems. Collecting neural signals is entirely dependent on the individuals, hence users must be very active during signal collection [105]. Nevertheless, there are now more studies focusing on neural signal processing to help paralyzed patients. Silent speech and handwriting recognition with the help of neural signals can be very useful for individuals with limitations in their speech and handwriting. Furthermore, these neural signals have the potential to pave the way for the development of advanced AR/VR applications in the near future. This review can be a great help to those interested in speech and handwriting recognition using neural signals.

## Figures and Tables

**Table 1:** Summary Table for features of brain signals.

Types of Neural Signal	Regions from Which Signals Are Acquired	Features Used in the Existing Reseach
EEG	Non-invasively from the scalp	Standard Deviation, Root Mean Square, Sum of values, and Energy of neural signals acquired at 128 Hz using Emotiv EPOC+ headset [2,67]. Fast Fourier transform and noise filtered signal extracted at 62.5–125 Hz using 40 channels EEG headset [2]. The energy of each frame neural signal was acquired at 250 Hz using EEG head cap [68]. EEG-acoustic features [69]. Feature descriptors such as Pyramid histogram of orientation gradients extracted at 128 Hz using Emotiv EPOC+ headset [78]. Discrete Fourier Transform and dynamic signature features extracted at 128 Hz using Emotiv EPOC+ headset [79]. Independent component analysis result of neural signals acquired at 250 Hz using 32 channel EEG electrode [80].
ECoG	Invasively from inside the skull. Generally from the primary motor cortex area of the brain [51]	High gamma activity (70–150 Hz) from the auditory and sensorimotor cortex [73]. High-frequency components (70–150 Hz) are recorded from the peri-Sylvian cortices [74]. Articulatory kinematic features from neural activity such as high gamma activity (70–200 Hz) and Low-frequency signal (1–30 Hz) features are recorded from ventral sensorimotor cortex [72,75]. High gamma frequency (65–250 Hz), mu frequency (7–13 Hz) and beta (15–30 Hz) frequency are recorded from cortex, frontal and temporal areas of brain [70]. Spatiotempral feature by time warping the acquired neural signal from premotor cortex [81].

**Table 2:** Summary for articles that focus on speech and handwritten recognition using neural signals.

Article	Feature Extraction, Methods and Results	Dataset Description (Invasive/Non-Invasive	Limitations
Kumar et al. [67]	Standard Deviation, Root Mean Square, Sum of values, Energy. Fine-level classification accuracy of 57.11% was achieved using the RF classifier	30 text and non-text class objects. 23 participants aged between 15 and 40 years. (Non-Invasive)	Fine level classification accuracy is not up to the mark
Rosinová et al. [68]	Feature vectors consisting of each frame’s energy. Very low accuracy using the HMM and GMM	50 voice commands from 20 participants (Non-Invasive)	Limited recording data and low accuracy
Krishna et al. [69]	EEG features, acoustic features and combination of EEG-acoustic features. A high recognition accuracy of 99.38% in the presence of background noise using GRU	Four English words—“yes”, “no”, “left”, and “right” spoken by 4 different people (Non-Invasive)	Limited variations in the dataset
Kapur et al. [35]	24-bit analog to digital converter sampled at 250 Hz. 81% accuracy, and information transfer rate of 203.73 bits per minute using CNN	10 trials of 15 sentences from three multiple sclerosis patients (Non-Invasive)	Limited variations in the dataset
Voront-sova et al. [2]	EEG features. 85% accuracy rate for the classification using ResNet18 and GRU	Nine Russian words as silent speech from 268 healthy participants (Non-Invasive)	Out-of-sample accuracy is relatively low in this study
Mugler et al. [70]	Spatiotemporal features. 36% accuracy in classifying phonemes with LDA	Entire set of phonemes from American English from 4 people (Invasive)	Only 18.8% accuracy in word identification from phonemic analysis
Anuman-chipalli et al. [72]	Acoustic features, articulatory kinematic features, spectral features. BLSTM has been used for decoding kinematic representations of articulation	High-density ECoG signals collected from 5 individuals (Invasive)	Experimental results are not discussed briefly
Moses et al. [73]	High gamma activity. Viterbi decoding was used with 61% decoding accuracy for producing utterances and 76% decoding accuracy for perceiving utterances.	ECoG recordings of 9 questions and 24 possible answers collected from 3 individuals (Invasive)	Limited variations in dataset
Makin et al. [74]	High frequency components. RNN used for training	30–50 sentences of data. 4 participants (Invasive)	Limited variations in dataset
Metzger et al. [75]	High gamma activity and Low frequency signal features. 6.13% character error rate and 29.4 characters per minute with GRU	NATO phonetic alphabet was used during spelling. 1 participant. (Invasive)	Only one participant was involved for training process
Chen et al. [77]	Filter bank analysis method. Spelling rate of up to 60 characters per minute with JFPM and decoding algorithm	Six blocks of 40 characters by 18 people (Non-Invasive)	Limited character sets
Saini et al. [78]	Pyramid histogram of orientation gradients features. 98.24%-person identification accuracy has been obtained using HMM classifiers	1400 samples of signatures and EEG signals. 70 participants. (Non-Invasive)	User verification results have not discussed briefly
Kumar et al. [79]	Dynamic signature features. 98.78% accuracy has been obtained by signature-EEG fusion data using BLSTM-NN classifiers	1980 samples of dynamic signatures and EEG signals from 58 users (Non-Invasive)	No. of samples for actual users are limited
Pei et al. [80]	Kinematic features. The accuracy of handwritten character recognition varied among participants, from 76.8% to 97% and cross-participant from 11.1% to 60% using CNN based classifiers	HELLO, WORLD! phrase by 5 participants (Non-Invasive)	Dataset is small and cross participant’s accuracy is low
Willett et al. [81]	Spatiotemporal features. 90 characters per minute decoding rate with 94.1% raw accuracy in real-time and greater than 99% accuracy offline using RNN	1000 handwriting sentences of 43,501 characters. 1 participant. (Invasive)	Ignored capital letters and text deletion and editing is not allowed

## Data Availability

Not applicable.

## Abbreviations

The following abbreviations are used in this manuscript:

MDPI	Multidisciplinary Digital Publishing Institute
BCI	Brain–Computer Interface
EEG	Electroencephalogram
ECoG	Electrocorticogram
LFP	Local Field Potential
CNN	Convolutional Neural Network
RNN	Recurrent Neural Network
LSTM	Long Short Term Memory
HMM	Hidden Markov Model
GMM	Gaussian Mixture Model
RF	Random Forest
LDA	Linear Discriminant Analysis
SSVEPs	Steady State Visual Evoked Potentials
GRU	Gated Recurrent Unit
BLSTM	Bidirectional Long Short Term Memory
ResNet	Residual Networks
EMG	Electromyography
sEMG	Surface Electromyography
IoT	Internet of Things
MFCC	Mel-frequency cepstral coefficient
BCI	Brain–Computer Interface
NATO	North Atlantic Treaty Organization
AR	Augmented Reality
SNR	Signal to Noise ratio
ANN	Artificial Neural Network
BMI	Brain Machine Interface
VR	Virtual Reality

## References

[1] Kübler A., Furdea A., Halder S., Hammer E.M., Nijboer F., Kotchoubey B. (2009). A brain–computer interface controlled auditory event-related potential (P300) spelling system for locked-in patients. Ann. N. Y. Acad. Sci..

[2] Vorontsova D., Menshikov I., Zubov A., Orlov K., Rikunov P., Zvereva E., Flitman L., Lanikin A., Sokolova A., Markov S. (2021). Silent eeg-speech recognition using convolutional and recurrent neural network with 85% accuracy of 9 words classification. Sensors.

[3] Santhanam G., Ryu S.I., Yu B.M., Afshar A., Shenoy K.V. (2006). A high-performance brain–computer interface. Nature.

[4] Rusnac A.L., Grigore O. (2022). CNN Architectures and Feature Extraction Methods for EEG Imaginary Speech Recognition. Sensors.

[5] Herff C., Schultz T. (2016). Automatic speech recognition from neural signals: A focused review. Front. Neurosci..

[6] Horlings R., Datcu D., Rothkrantz L.J. Emotion recognition using brain activity. Proceedings of the 9th International Conference on Computer Systems and Technologies and Workshop for PhD Students in Computing.

[7] Patil A., Deshmukh C., Panat A. Feature extraction of EEG for emotion recognition using Hjorth features and higher order crossings. Proceedings of the 2016 Conference on Advances in Signal Processing (CASP).

[8] Lotte F. (2014). A tutorial on EEG signal-processing techniques for mental-state recognition in brain–computer interfaces. Guide to Brain-Computer Music Interfacing.

[9] Brigham K., Kumar B.V. Subject identification from electroencephalogram (EEG) signals during imagined speech. Proceedings of the 2010 Fourth IEEE International Conference on Biometrics: Theory, Applications and Systems (BTAS).

[10] Mirkovic B., Bleichner M.G., De Vos M., Debener S. (2016). Target speaker detection with concealed EEG around the ear. Front. Neurosci..

[11] Brumberg J.S., Nieto-Castanon A., Kennedy P.R., Guenther F.H. (2010). Brain–computer interfaces for speech communication. Speech Commun..

[12] Soman S., Murthy B. (2015). Using brain computer interface for synthesized speech communication for the physically disabled. Procedia Comput. Sci..

[13] Ahn M., Lee M., Choi J., Jun S.C. (2014). A review of brain–computer interface games and an opinion survey from researchers, developers and users. Sensors.

[14] Sadeghi K., Banerjee A., Sohankar J., Gupta S.K. Optimization of brain mobile interface applications using IoT. Proceedings of the 2016 IEEE 23rd International Conference on High Performance Computing (HiPC).

[15] Eleryan A., Vaidya M., Southerland J., Badreldin I.S., Balasubramanian K., Fagg A.H., Hatsopoulos N., Oweiss K. (2014). Tracking single units in chronic, large scale, neural recordings for brain machine interface applications. Front. Neuroeng..

[16] Sussillo D., Stavisky S.D., Kao J.C., Ryu S.I., Shenoy K.V. (2016). Making brain–machine interfaces robust to future neural variability. Nat. Commun..

[17] Lebedev M.A., Nicolelis M.A. (2006). Brain–machine interfaces: Past, present and future. Trends Neurosci..

[18] Vázquez-Guardado A., Yang Y., Bandodkar A.J., Rogers J.A. (2020). Recent advances in neurotechnologies with broad potential for neuroscience research. Nat. Neurosci..

[19] Illes J., Moser M.A., McCormick J.B., Racine E., Blakeslee S., Caplan A., Hayden E.C., Ingram J., Lohwater T., McKnight P. (2010). Neurotalk: Improving the communication of neuroscience research. Nat. Rev. Neurosci..

[20] Koct M., Juh J. Speech Activity Detection from EEG using a feed-forward neural network. Proceedings of the 2019 10th IEEE International Conference on Cognitive Infocommunications (CogInfoCom).

[21] Koctúrová M., Juhár J. (2021). A Novel approach to EEG speech activity detection with visual stimuli and mobile BCI. Appl. Sci..

[22] Gannouni S., Aledaily A., Belwafi K., Aboalsamh H. (2021). Emotion detection using electroencephalography signals and a zero-time windowing-based epoch estimation and relevant electrode identification. Sci. Rep..

[23] Luo S., Rabbani Q., Crone N.E. (2022). Brain-computer interface: Applications to speech decoding and synthesis to augment communication. Neurotherapeutics.

[24] Pandarinath C., Nuyujukian P., Blabe C.H., Sorice B.L., Saab J., Willett F.R., Hochberg L.R., Shenoy K.V., Henderson J.M. (2017). High performance communication by people with paralysis using an intracortical brain–computer interface. elife.

[25] Stavisky S.D., Willett F.R., Wilson G.H., Murphy B.A., Rezaii P., Avansino D.T., Memberg W.D., Miller J.P., Kirsch R.F., Hochberg L.R. (2019). Neural ensemble dynamics in dorsal motor cortex during speech in people with paralysis. elife.

[26] Gorno-Tempini M.L., Hillis A.E., Weintraub S., Kertesz A., Mendez M., Cappa S.F., Ogar J.M., Rohrer J.D., Black S., Boeve B.F. (2011). Classification of primary progressive aphasia and its variants. Neurology.

[27] Willett F.R., Murphy B.A., Memberg W.D., Blabe C.H., Pandarinath C., Walter B.L., Sweet J.A., Miller J.P., Henderson J.M., Shenoy K.V. (2017). Signal-independent noise in intracortical brain–computer interfaces causes movement time properties inconsistent with Fitts’ law. J. Neural Eng..

[28] Brumberg J.S., Wright E.J., Andreasen D.S., Guenther F.H., Kennedy P.R. (2011). Classification of intended phoneme production from chronic intracortical microelectrode recordings in speech motor cortex. Front. Neurosci..

[29] Rabbani Q., Milsap G., Crone N.E. (2019). The potential for a speech brain–computer interface using chronic electrocorticography. Neurotherapeutics.

[30] Yang T., Hakimian S., Schwartz T.H. (2014). Intraoperative ElectroCorticoGraphy (ECog): Indications, techniques, and utility in epilepsy surgery. Epileptic Disord..

[31] Kirschstein T., Köhling R. (2009). What is the source of the EEG?. Clin. EEG Neurosci..

[32] Casson A.J., Smith S., Duncan J.S., Rodriguez-Villegas E. Wearable EEG: What is it, why is it needed and what does it entail?. Proceedings of the 2008 30th Annual International Conference of the IEEE Engineering in Medicine and Biology Society.

[33] Tandra R., Sahai A. (2008). SNR walls for signal detection. IEEE J. Sel. Top. Signal Process..

[34] Wilson G.H., Stavisky S.D., Willett F.R., Avansino D.T., Kelemen J.N., Hochberg L.R., Henderson J.M., Druckmann S., Shenoy K.V. (2020). Decoding spoken English from intracortical electrode arrays in dorsal precentral gyrus. J. Neural Eng..

[35] Kapur A., Sarawgi U., Wadkins E., Wu M., Hollenstein N., Maes P. Non-invasive silent speech recognition in multiple sclerosis with dysphonia. Proceedings of the Machine Learning for Health Workshop. PMLR.

[36] Müller-Putz G.R., Scherer R., Brauneis C., Pfurtscheller G. (2005). Steady-state visual evoked potential (SSVEP)-based communication: Impact of harmonic frequency components. J. Neural Eng..

[37] Chandler J.A., Van der Loos K.I., Boehnke S., Beaudry J.S., Buchman D.Z., Illes J. (2022). Brain Computer Interfaces and Communication Disabilities: Ethical, legal, and social aspects of decoding speech from the brain. Front. Hum. Neurosci..

[38] What Part of the Brain Controls Speech?. https://www.healthline.com/health/what-part-of-the-brain-controls-speech.

[39] The Telltale Hand. https://www.dana.org/article/the-telltale-hand/#:~:text=The%20sequence%20that%20produces%20handwriting,content%20of%20the%20motor%20sequence.

[40] How Does Your Brain Control Speech?. https://districtspeech.com/how-does-your-brain-control-speech/.

[41] Obleser J., Wise R.J., Dresner M.A., Scott S.K. (2007). Functional integration across brain regions improves speech perception under adverse listening conditions. J. Neurosci..

[42] What Part of the Brain Controls Speech? Brain Hemispheres Functions REGIONS of the Brain Brain Injury and Speech. https://psychcentral.com/health/what-part-of-the-brain-controls-speech.

[43] Chang E.F., Rieger J.W., Johnson K., Berger M.S., Barbaro N.M., Knight R.T. (2010). Categorical speech representation in human superior temporal gyrus. Nat. Neurosci..

[44] Willett F.R., Deo D.R., Avansino D.T., Rezaii P., Hochberg L.R., Henderson J.M., Shenoy K.V. (2020). Hand knob area of premotor cortex represents the whole body in a compositional way. Cell.

[45] James K.H., Engelhardt L. (2012). The effects of handwriting experience on functional brain development in pre-literate children. Trends Neurosci. Educ..

[46] Palmis S., Danna J., Velay J.L., Longcamp M. (2017). Motor control of handwriting in the developing brain: A review. Cogn. Neuropsychol..

[47] Neural Prosthesis Uses Brain Activity to Decode Speech. https://medicalxpress.com/news/2023-01-neural-prosthesis-brain-decode-speech.html.

[48] Maas A.I., Harrison-Felix C.L., Menon D., Adelson P.D., Balkin T., Bullock R., Engel D.C., Gordon W., Langlois-Orman J., Lew H.L. (2011). Standardizing data collection in traumatic brain injury. J. Neurotrauma.

[49] Difference between Invasive and Non-Invasive BCI|Types of BCIs. https://www.rfwireless-world.com/Terminology/Difference-between-BCI-types.html.

[50] What Is a Brain-Computer Interface? Everything You Need to Know about BCIs, Neural Interfaces and the Future of Mind-Reading Computers. https://www.zdnet.com/article/what-is-bci-everything-you-need-to-know-about-brain-computer-interfaces-and-the-future-of-mind-reading-computers/.

[51] Downey J.E., Schwed N., Chase S.M., Schwartz A.B., Collinger J.L. (2018). Intracortical recording stability in human brain–computer interface users. J. Neural Eng..

[52] Hochberg L.R., Bacher D., Jarosiewicz B., Masse N.Y., Simeral J.D., Vogel J., Haddadin S., Liu J., Cash S.S., Van Der Smagt P. (2012). Reach and grasp by people with tetraplegia using a neurally controlled robotic arm. Nature.

[53] Chakrabarti S., Sandberg H.M., Brumberg J.S., Krusienski D.J. (2015). Progress in speech decoding from the electrocorticogram. Biomed. Eng. Lett..

[54] Herff C., Heger D., De Pesters A., Telaar D., Brunner P., Schalk G., Schultz T. (2015). Brain-to-text: Decoding spoken phrases from phone representations in the brain. Front. Neurosci..

[55] Bouchard K.E., Chang E.F. Neural decoding of spoken vowels from human sensory-motor cortex with high-density electrocorticography. Proceedings of the 2014 36th Annual International Conference of the IEEE Engineering in Medicine and Biology Society.

[56] Heger D., Herff C., Pesters A.D., Telaar D., Brunner P., Schalk G., Schultz T. Continuous speech recognition from ECOG. Proceedings of the Sixteenth Annual Conference of the International Speech Communication Association.

[57] Miniussi C., Harris J.A., Ruzzoli M. (2013). Modelling non-invasive brain stimulation in cognitive neuroscience. Neurosci. Biobehav. Rev..

[58] Data Augmentation for Brain-Computer Interface. https://towardsdatascience.com/data-augmentation-for-brain-computer-interface-35862c9beb40.

[59] Grau C., Ginhoux R., Riera A., Nguyen T.L., Chauvat H., Berg M., Amengual J.L., Pascual-Leone A., Ruffini G. (2014). Conscious brain-to-brain communication in humans using non-invasive technologies. PLoS ONE.

[60] Porbadnigk A., Wester M., Calliess J.P., Schultz T. EEG-based speech recognition. Proceedings of the BIOSIGNALS 2009—International Conference on Bio-Inspired Systems and Signal Processing.

[61] Jiménez-Guarneros M., Gómez-Gil P. (2021). Standardization-refinement domain adaptation method for cross-subject EEG-based classification in imagined speech recognition. Pattern Recognit. Lett..

[62] Kumar P., Scheme E. A deep spatio-temporal model for EEG-based imagined speech recognition. Proceedings of the ICASSP 2021-2021 IEEE International Conference on Acoustics, Speech and Signal Processing (ICASSP).

[63] Al-Kadi M.I., Reaz M.B.I., Ali M.A.M. (2013). Evolution of electroencephalogram signal analysis techniques during anesthesia. Sensors.

[64] Kakigi R., Inui K., Tran D.T., Qiu Y., Wang X., Watanabe S., Hoshiyama M. (2004). Human brain processing and central mechanisms of pain as observed by electro-and magneto-encephalography. J.-Chin. Med. Assoc..

[65] Ogawa S., Menon R., Kim S.G., Ugurbil K. (1998). On the characteristics of functional magnetic resonance imaging of the brain. Annu. Rev. Biophys. Biomol. Struct..

[66] Brain-Computer Interfaces. https://cs181-bcis.weebly.com/non-invasive-bcis.html#:~:text=What%20is%20a%20%22non-invasive%20BCI%3F%22%20The%20term%20%E2%80%9Cnon-invasive,brain-to-computer%20stimulation%20without%20needing%20to%20penetrate%20the%20skull.

[67] Kumar P., Saini R., Roy P.P., Sahu P.K., Dogra D.P. (2018). Envisioned speech recognition using EEG sensors. Pers. Ubiquitous Comput..

[68] Rosinová M., Lojka M., Staš J., Juhár J. Voice command recognition using eeg signals. Proceedings of the 2017 International Symposium ELMAR.

[69] Krishna G., Tran C., Yu J., Tewfik A.H. Speech recognition with no speech or with noisy speech. Proceedings of the ICASSP 2019-2019 IEEE International Conference on Acoustics, Speech and Signal Processing (ICASSP).

[70] Mugler E.M., Patton J.L., Flint R.D., Wright Z.A., Schuele S.U., Rosenow J., Shih J.J., Krusienski D.J., Slutzky M.W. (2014). Direct classification of all American English phonemes using signals from functional speech motor cortex. J. Neural Eng..

[71] Moses D.A., Mesgarani N., Leonard M.K., Chang E.F. (2016). Neural speech recognition: Continuous phoneme decoding using spatiotemporal representations of human cortical activity. J. Neural Eng..

[72] Anumanchipalli G.K., Chartier J., Chang E.F. (2019). Speech synthesis from neural decoding of spoken sentences. Nature.

[73] Moses D.A., Leonard M.K., Makin J.G., Chang E.F. (2019). Real-time decoding of question-and-answer speech dialogue using human cortical activity. Nat. Commun..

[74] Makin J.G., Moses D.A., Chang E.F. (2020). Machine translation of cortical activity to text with an encoder–decoder framework. Nat. Neurosci..

[75] Metzger S.L., Liu J.R., Moses D.A., Dougherty M.E., Seaton M.P., Littlejohn K.T., Chartier J., Anumanchipalli G.K., Tu-Chan A., Ganguly K. (2022). Generalizable spelling using a speech neuroprosthesis in an individual with severe limb and vocal paralysis. Nat. Commun..

[76] NATO Phonetic Alphabet. https://first10em.com/quick-reference/nato-phonetic-alphabet/#:~:text=Alpha%2C%20Bravo%2C%20Charlie%2C%20Delta,%2Dray%2C%20Yankee%2C%20Zulu.

[77] Chen X., Wang Y., Nakanishi M., Gao X., Jung T.P., Gao S. (2015). High-speed spelling with a noninvasive brain–computer interface. Proc. Natl. Acad. Sci. USA.

[78] Saini R., Kaur B., Singh P., Kumar P., Roy P.P., Raman B., Singh D. (2018). Don’t just sign use brain too: A novel multimodal approach for user identification and verification. Inf. Sci..

[79] Kumar P., Saini R., Kaur B., Roy P.P., Scheme E. (2019). Fusion of neuro-signals and dynamic signatures for person authentication. Sensors.

[80] Pei L., Ouyang G. (2021). Online recognition of handwritten characters from scalp-recorded brain activities during handwriting. J. Neural Eng..

[81] Willett F.R., Avansino D.T., Hochberg L.R., Henderson J.M., Shenoy K.V. (2021). High-performance brain-to-text communication via handwriting. Nature.

[82] Saby J.N., Marshall P.J. (2012). The utility of EEG band power analysis in the study of infancy and early childhood. Dev. Neuropsychol..

[83] Dubey A., Ray S. (2020). Comparison of tuning properties of gamma and high-gamma power in local field potential (LFP) versus electrocorticogram (ECoG) in visual cortex. Sci. Rep..

[84] Saeidi M., Karwowski W., Farahani F.V., Fiok K., Taiar R., Hancock P., Al-Juaid A. (2021). Neural decoding of EEG signals with machine learning: A systematic review. Brain Sci..

[85] Agarwal P., Kumar S. (2022). Electroencephalography-based imagined speech recognition using deep long short-term memory network. ETRI J..

[86] Angrick M., Herff C., Mugler E., Tate M.C., Slutzky M.W., Krusienski D.J., Schultz T. (2019). Speech synthesis from ECoG using densely connected 3D convolutional neural networks. J. Neural Eng..

[87] Hinton G., Deng L., Yu D., Dahl G.E., Mohamed A.R., Jaitly N., Senior A., Vanhoucke V., Nguyen P., Sainath T.N. (2012). Deep neural networks for acoustic modeling in speech recognition: The shared views of four research groups. IEEE Signal Process. Mag..

[88] Akar S.A., Kara S., Agambayev S., Bilgiç V. Nonlinear analysis of EEG in major depression with fractal dimensions. Proceedings of the 2015 37th Annual International Conference of the IEEE Engineering in Medicine and Biology Society (EMBC).

[89] Nieto N., Peterson V., Rufiner H.L., Kamienkowski J.E., Spies R. (2022). Thinking out loud, an open-access EEG-based BCI dataset for inner speech recognition. Sci. Data.

[90] Alqatawneh A., Alhalaseh R., Hassanat A., Abbadi M. (2019). Statistical-hypothesis-aided tests for epilepsy classification. Computers.

[91] Chenane K., Touati Y., Boubchir L., Daachi B. (2019). Neural net-based approach to EEG signal acquisition and classification in BCI applications. Computers.

[92] Borghini G., Aricò P., Di Flumeri G., Sciaraffa N., Babiloni F. (2019). Correlation and similarity between cerebral and non-cerebral electrical activity for user’s states assessment. Sensors.

[93] Lim J.Z., Mountstephens J., Teo J. (2020). Emotion recognition using eye-tracking: Taxonomy, review and current challenges. Sensors.

[94] Hughes A., Jorda S. (2021). Applications of Biological and Physiological Signals in Commercial Video Gaming and Game Research: A Review. Front. Comput. Sci..

[95] Li M., Chen W., Zhang T. (2017). Classification of epilepsy EEG signals using DWT-based envelope analysis and neural network ensemble. Biomed. Signal Process. Control..

[96] Li X., Samuel O.W., Zhang X., Wang H., Fang P., Li G. (2017). A motion-classification strategy based on sEMG-EEG signal combination for upper-limb amputees. J. Neuroeng. Rehabil..

[97] Yang S., Li M., Wang J. (2022). Fusing sEMG and EEG to Increase the Robustness of Hand Motion Recognition Using Functional Connectivity and GCN. IEEE Sens. J..

[98] Zhang X., Ma Z., Zheng H., Li T., Chen K., Wang X., Liu C., Xu L., Wu X., Lin D. (2020). The combination of brain–computer interfaces and artificial intelligence: Applications and challenges. Ann. Transl. Med..

[99] Pei D., Vinjamuri R. (2020). Introductory chapter: Methods and applications of neural signal processing. Advances in Neural Signal Processing.

[100] Taplin A.M., de Pesters A., Brunner P., Hermes D., Dalfino J.C., Adamo M.A., Ritaccio A.L., Schalk G. (2016). Intraoperative mapping of expressive language cortex using passive real-time electrocorticography. Epilepsy Behav. Case Rep..

[101] Hill N.J., Gupta D., Brunner P., Gunduz A., Adamo M.A., Ritaccio A., Schalk G. (2012). Recording human electrocorticographic (ECoG) signals for neuroscientific research and real-time functional cortical mapping. JoVE (J. Vis. Exp.).

[102] Jeong J.H., Shim K.H., Kim D.J., Lee S.W. (2020). Brain-controlled robotic arm system based on multi-directional CNN-BiLSTM network using EEG signals. IEEE Trans. Neural Syst. Rehabil. Eng..

[103] Burwell S., Sample M., Racine E. (2017). Ethical aspects of brain computer interfaces: A scoping review. BMC Med. Ethics.

[104] Glannon W. (2014). Ethical Issues with Brain-Computer Interfaces. Front. Syst. Neurosci..

[105] Mridha M.F., Das S.C., Kabir M.M., Lima A.A., Islam M.R., Watanobe Y. (2021). Brain-computer interface: Advancement and challenges. Sensors.

[106] Saha S., Mamun K.A., Ahmed K., Mostafa R., Naik G.R., Darvishi S., Khandoker A.H., Baumert M. (2021). Progress in brain computer interface: Challenges and opportunities. Front. Syst. Neurosci..

[107] Chatterjee B., Nath M., Xiao S., Jayant K., Sen S. (2022). Bi-Phasic Quasistatic Brain Communication for Fully Untethered Connected Brain Implants. bioRxiv.

[108] Chatterjee B., Kumar G., Nath M., Xiao S., Modak N., Das D., Krishna J., Sen S. A 1.15 μW 5.54 mm^3^ implant with a bidirectional neural sensor and stimulator SoC utilizing bi-phasic quasi-static brain communication achieving 6 kbps–10 Mbps uplink with compressive sensing and RO-PUF based collision avoidance. Proceedings of the 2021 Symposium on VLSI Circuits.

[109] Chatterjee B., Kumar K.G., Xiao S., Barik G., Jayant K., Sen S. A 1.8 μW 5.5 mm^3^ ADC-less Neural Implant SoC utilizing 13.2 pJ/Sample Time-domain Bi-phasic Quasi-static Brain Communication with Direct Analog to Time Conversion. Proceedings of the ESSCIRC 2022-IEEE 48th European Solid State Circuits Conference (ESSCIRC).

[110] Khalifa A., Liu Y., Karimi Y., Wang Q., Eisape A., Stanaćević M., Thakor N., Bao Z., Etienne-Cummings R. (2019). The microbead: A 0.009 mm^3^ implantable wireless neural stimulator. IEEE Trans. Biomed. Circuits Syst..

[111] Khalifa A., Karimi Y., Wang Q., Montlouis W., Garikapati S., Stanaćević M., Thakor N., Etienne-Cummings R. (2018). The microbead: A highly miniaturized wirelessly powered implantable neural stimulating system. IEEE Trans. Biomed. Circuits Syst..

